# Case of Lumbrici in the Cavity of the Peritoneum

**Published:** 1839-02-02

**Authors:** N. L. Thomas

**Affiliations:** Tennessee


*Case of Lumbrici in the Cavity of the Peritoneum.* By N. L. Thomas, M. D., of Tennessee.

I was, in 1836, called to see a negro child, about three years old, labouring under the usual symptoms of enteritis. The disease was so far advanced that no remedies produced any effect, and the child died in about twelve hours.

*Autopsy.*—The small intestines showed very marked inflammation; but the interesting part of the examination was the discovery of eight lumbrici in the cavity of the abdomen. Five of them were of the largest size, the other three small. They were almost entirely entangled in a tough mucus. After removing them from the surface of the liver, a track was seen similar to that left by the common earth worm in passing over very soft mud. At least half of their circumference had been bedded in the liver, producing, however, no other altered appearance in its surface. No perforation could be found in any portion of the intestinal canal. I presume, from the track they had made in the liver, that their escape from the intestines, (if it had occurred at all,) was not of recent date. There were only two worms in the cavity of the tube. It may be a question of some interest to inquire, how far these worms were active in producing the enteritis. There are, however, no means of ascertaining this fact; and I shall leave each one to draw his own conclusions, having stated the facts.

